# Infrared thermometer on the wall (iThermowall): An open source and 3-D print infrared thermometer for fever screening

**DOI:** 10.1016/j.ohx.2020.e00168

**Published:** 2020-12-26

**Authors:** Tomy Abuzairi, Nur Imaniati Sumantri, Ahli Irfan, Ridho Maulana Mohamad

**Affiliations:** a Electrical Engineering, Department of Electrical Engineering, Faculty of Engineering, Universitas Indonesia, Depok 16424, Indonesia; b Biomedical Engineering, Department of Electrical Engineering, Faculty of Engineering, Universitas Indonesia, Depok 16424, Indonesia

**Keywords:** Thermometer, Infrared, Fever screening, Open-source, 3-D print

## Abstract

In this COVID-19 pandemic, a non-contact handheld infrared thermometer is frequently used for fever screening. However, the handheld thermometer performance depends on the operator and the distance to the forehead. To address these problems, we present an infrared thermometer on the wall (iThermowall). The iThermowall is a low-cost non-contact thermometer, adapted for the use of fever screening in public areas without an operator. The hardware can measure human body temperature automatically when the distance between the sensor and forehead is adequate. Temperature measurement validation of the iThermowall was conducted by T-test analysis. The results show that the P-values for all the test is more significant than 0.05, means that the mean Celsius temperature for both groups (reference thermometer and iThermowall) are similar. This article provides the 3-D printable open-source and the full source code firmware for the developing and under-resourced communities.

## Graphical abstract

**Figure:**
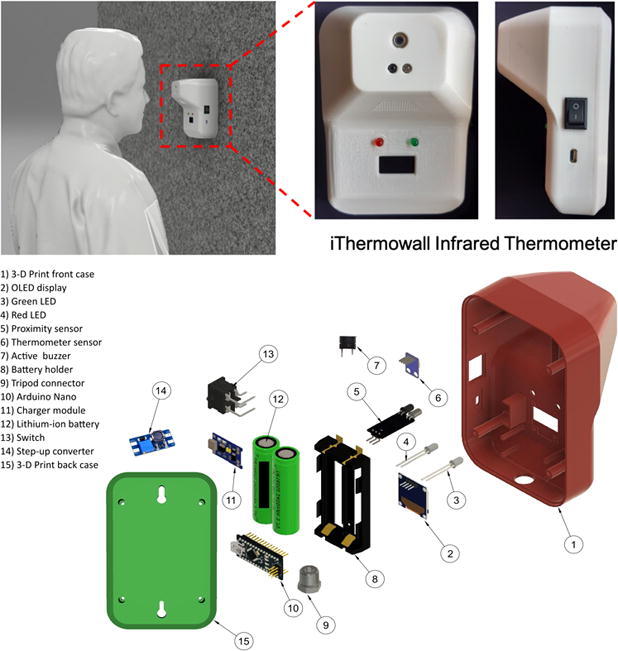

## 1 Specifications table

**Table:** 

Hardware name	iThermowall Thermometer
Subject area	Medical
Hardware type	Medical hardware
Open Source License	GNU General Public License (GPL) v3.0
Cost of Hardware	$35 USD
Source File Repository	http://doi.org/10.5281/zenodo.4127545

## 2 Hardware in context

Severe acute respiratory syndrome coronavirus 2 (SARS-CoV-2) has been spread widely around the world through the airborne mechanism. As of October 4, 2020, the global cumulative cases of coronavirus is 34.8 million cases [1]. This pathogen interacts and survives in the human body primarily by attaching its spike protein to the angiotensin-converting enzyme 2 (ACE2), especially expressed in the alveoli, to multiply its genetic material and produce new viruses. The disease resulted from SARS-CoV-2 infection is called coronavirus disease 2019 (COVID-19). Disease onset appears as SARS-CoV-2 multiply in the lung; alveolar damage is developed, leading to progressive respiratory [2].

There are commonly four types of COVID-19 patients: asymptomatic, mild, moderate, and severe [3]. Fever, dry cough, diarrhea, breathing difficulties (dyspnea), headache, and pneumonia are the most frequent symptoms of COVID-19. Monitoring body temperature is essential, especially for the early detection of COVID-19 suspects. The variation of individuals’ temperature is influenced by several factors, such as gender and age. However, the baseline body temperature should be used to characterize the threshold temperature for fever [4]. The temperature interferes virus viability. SARS-CoV-2 can be stable in infected patients for up to 4 days [5]. This viral stability at temperatures around normal body temperature implies that temperature may play a particularly significant role in the transmission and severity of COVID-19. The fluctuations around 37 °C may interrupt viral stability. Viral viability was rapidly lost at higher temperatures and higher relative humidity (e.g., 38 °C, and relative humidity of >95%) [6]. Lower body temperatures may bolster expeditious viral growth that further correlated with disease progression.

Generally, fever screening is conducted at a place where lots of people gather and have the potential to transmit the virus, such as hospitals and airports [7], [8]. Fever screening in the public area was proven to help early detection in several viral outbreaks, such as dengue virus and Ebola, hence gave a positive effect on partially blocking the importation of cases [9]. Clinical electronic thermometers are an effective screening and indicative device to aid the recognizable proof of those people who might be tainted with COVID-19. The guidance represented by Food and Drug Administration helps address these earnest public health worries by assisting with extending the accessibility of clinical electronic thermometers during this crisis, thereby helping to prevent potential shortages as the demand increases due to usage at critical locations [10].

In this COVID-19 pandemic, a non-contact handheld infrared thermometer is frequently used for fever screening. This thermometer has the advantage of being a fast, non-contact, and easy-to-use of fever screening [11]. However, the handheld thermometer performance depends on the operator and the distance to the forehead [12]. To address these obstacles, we introduce a non-contact infrared thermometer on the wall (iThermowall). The iThermowall is an open-source and low-cost platform thermometer for fever screening that does not require an operator. The hardware also can measure body temperature automatically when the distance between the sensor and forehead is adequate.

## 3 Hardware description

**Fig. 1:**
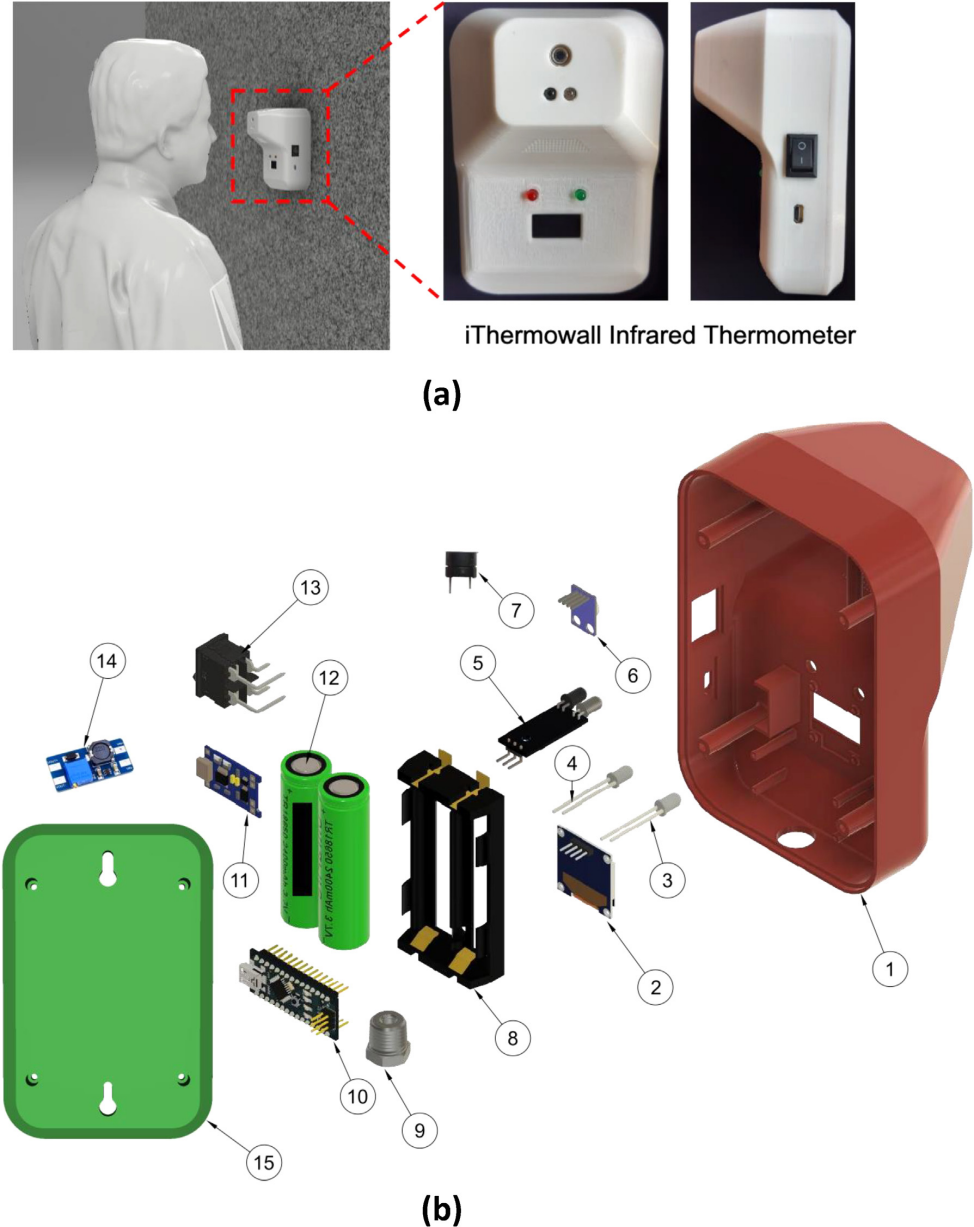
(a) Front-view and side-view of the iThermowall. (b) Detail parts of the thermometer: 1) front case, 2) OLED display, 3) green LED, 4) red LED, 5) infrared proximity sensor module, 6) infrared thermometer sensor module, 7) active buzzer, 8) battery holder, 9) tripod connector, 10) Arduino microcontroller, 11) charger module, 12) lithium-ion battery 18650, 13) switch, 14) step-up converter, 15) back case. (For interpretation of the references to colour in this figure legend, the reader is referred to the web version of this article.)

The iThermowall thermometer (Fig. 1a) was designed to be easily reproducible by using a readily accessible module and 3-D printer. Fig. 1b shows the detailed parts of the iThermowall. The electronic components of this hardware consist of a microcontroller unit (MCU), OLED display, LED, infrared thermometer sensor, infrared proximity sensor, buzzer, charger module, step-up converter, and battery.

The MCU utilized open-source Arduino Nano microcontrollers [13]. Arduino Nano uses an ATmega328 microcontroller with 32 KB of flash memory, 2 KB of SRAM, and 1 KB of EEPROM. Additionally, Arduino Nano is utilized besides its capability using I2C communication to read data from the infrared thermometer sensor and write to the OLED display. A step-up converter was utilized as a step-up voltage from 3.7 V to 5 V for powering the electronic component. Battery to power the hardware used two parallel Lithium-ion 18,650 with a capacity of 2200 mAh and voltage of 3.7 V. A charger module TP4056 can charge the lithium-ion battery with battery protection. The infrared thermometer sensor used the GY-906 module that utilized MLX90614 temperature sensor. This module sensor is easy to integrate with Arduino Nano because of I2C communication. To measure the distance between the forehead and hardware, an infrared proximity sensor module was utilized. The distance was set using a potentiometer inside the infrared proximity sensor module. The result of the temperature sensor was shown in the OLED SSD1306 with a size of 128 mm × 64 mm [14]. Green LED was used to show that the temperature is below 38 °C and Red LED was used to show that the temperature is above 38 °C. An active buzzer was utilized to warn for 5 s if the temperature sensor value is more than 38 °C. Finally, a switch was used to turn off the hardware. It will be very handy if the hardware wants to be moved to another place or when it is charged.

Different techniques are being used by many researchers to measure human body temperature. The measurements are performed with various types of sensors. Zakaria *et al.* used the LM35 sensor to measure infant body temperature [15]. Azizulkarim *et al.* utilized DS18D20 to monitor real-time patient temperature [16]. However, the weakness of LM35 and DS18B20 sensors for fever screening COVID-19 is that there must be contact with the sensor. For non-contact human body temperature, the MLX906 sensor is being used by many researchers [17], [18], [19], [20]. It can measure a wide temperature range: −40 to 125 °C for sensor temperature and −70 to 380 °C for object temperature [21]. Asif et al. used an MLX906 sensor to measure body temperature in real-time using IoT [18]. A contactless infrared thermometer prototype has been developed using Arduino Uno MCU with an ultrasonic sensor as distance sensor [22]. However, the prototype did not perform a thorough validation of the hardware and did not have provide a casing for the electronic components. Advanced human body temperature measurement could be conducted using a camera, such as FLIR Thermovision [23] and OptoTerm Thermoscreen [24]. The advantage of the camera sensor system is that it can measure many people's temperature quickly, while the price of the camera sensor system is costly (more than $ 15,000) [25].

In this hardware, we design iThermowall that is a low-cost and non-contact thermometer. The iThermowall can automatically measure human body temperature using a proximity sensor when the distance between the sensor and forehead is adequate. Researchers who use the iThermowall and system will do so because it offers:

• A simple assembly hardware with microcontroller, display, and sensor that are available as manufactured modules,
• An open-source file to program the thermometer system algorithm that can be modified with Arduino integrated development environment software,
• An open-source file to design the schematic and footprint of the circuit that can be modified.

## 4 Design files

**Table:** 

Design file name	Image	File type	Open source license	Location of the file
**iThermowall enclosure**				
FrontCase_iThermowall.stl		STL	GNU GPL v3	http://doi.org/10.5281/zenodo.4127545
BackCase_iThermowall.stl		STL	GNU GPL v3	http://doi.org/10.5281/zenodo.4127545

**iThermowall electronic schematic**				
Schematic_iThermowall.pdf	–	PDF	GNU GPL v3	http://doi.org/10.5281/zenodo.4127545
Schematic_iThermowall.json	–	JSON	GNU GPL v3	http://doi.org/10.5281/zenodo.4127545

**iThermowall firmware**				
iThermowall_firmware.ino	–	Arduino sketch	GNU GPL v3	http://doi.org/10.5281/zenodo.4127545
Adafruit_SSD1306.zip	–	OLED library	GNU GPL v3	http://doi.org/10.5281/zenodo.4127545
Adafruit_MLX90614.zip	–	Sensor library	GNU GPL v3	http://doi.org/10.5281/zenodo.4127545
millisDelay.zip	–	Delay library	GNU GPL v3	http://doi.org/10.5281/zenodo.4127545

### 4.1 Bill of materials

#### 4.1.1 Electronic system bill of materials

**Table:** 

**Designator**	**Component**	**Number**	**Cost per unit - USD**	**Total cost - USD**	**Source of materials**
U1	Arduino Nano ATMEGA328P	1	$4.50	$4.50	https://www.aliexpress.com/item/32605261942.html
U2	GY-906 Temperature Sensor Module	1	$9.5	$9.5	https://www.aliexpress.com/item/32739146147.html
U3	OLED SSD1306 128x64	1	$1.95	$1.95	https://www.aliexpress.com/item/32830523451.html
U4	Infrared Proximity Sensor Module	1	$0.38	$0.38	https://www.aliexpress.com/item/4000550056776.html
U5	TP4056 Lithium Battery Charger Module – Micro USB	1	$0.30	$0.30	https://www.aliexpress.com/item/32649780468.html
U6	Battery 18,650 Holder 2 port	1	$1.09	$1.09	https://www.aliexpress.com/item/32993381574.html
U7	5 V DC-DC Step Up Converter	1	$0.25	$0.25	https://www.aliexpress.com/item/1005001556639784.html
SW1	Switch 2 Pin 15 mm × 21 mm	1	$0.12	$0.12	https://www.aliexpress.com/item/33059866113.html
LED1	Green LED 5 mm	1	$0.01	$0.01	https://www.aliexpress.com/item/32840584788.html
LED2	Red LED 5 mm	1	$0.01	$0.01	https://www.aliexpress.com/item/32839689680.html
	Battery 18,650	2	$3.99	$7.98	https://www.aliexpress.com/item/4000735911349.html
	Jumper Dupont Wire Cable	1	$2.64	$2.64	https://www.aliexpress.com/item/33060775595.html

#### 4.1.2 Mechanical system bill of materials

**Table:** 

Designator	Component	Mass in kg	Cost per unit - USD	Total cost - USD	Source of materials	Material type
Case1	FrontCase_iThermowall.stl	0.146	$35.69 / kg	$5.211	https://www.aliexpress.com/item/1005001298882187.html	PLA + 1.75 mm – 3-D printing filament.
Case2	BackCase_iThermowall.stl	0.075	$35.69 / kg	$2.677	https://www.aliexpress.com/item/1005001298882187.html	PLA + 1.75 mm – 3-D printing filament.

## 5 Build instructions

### 5.1 3-D print casing

**Fig. 2:**
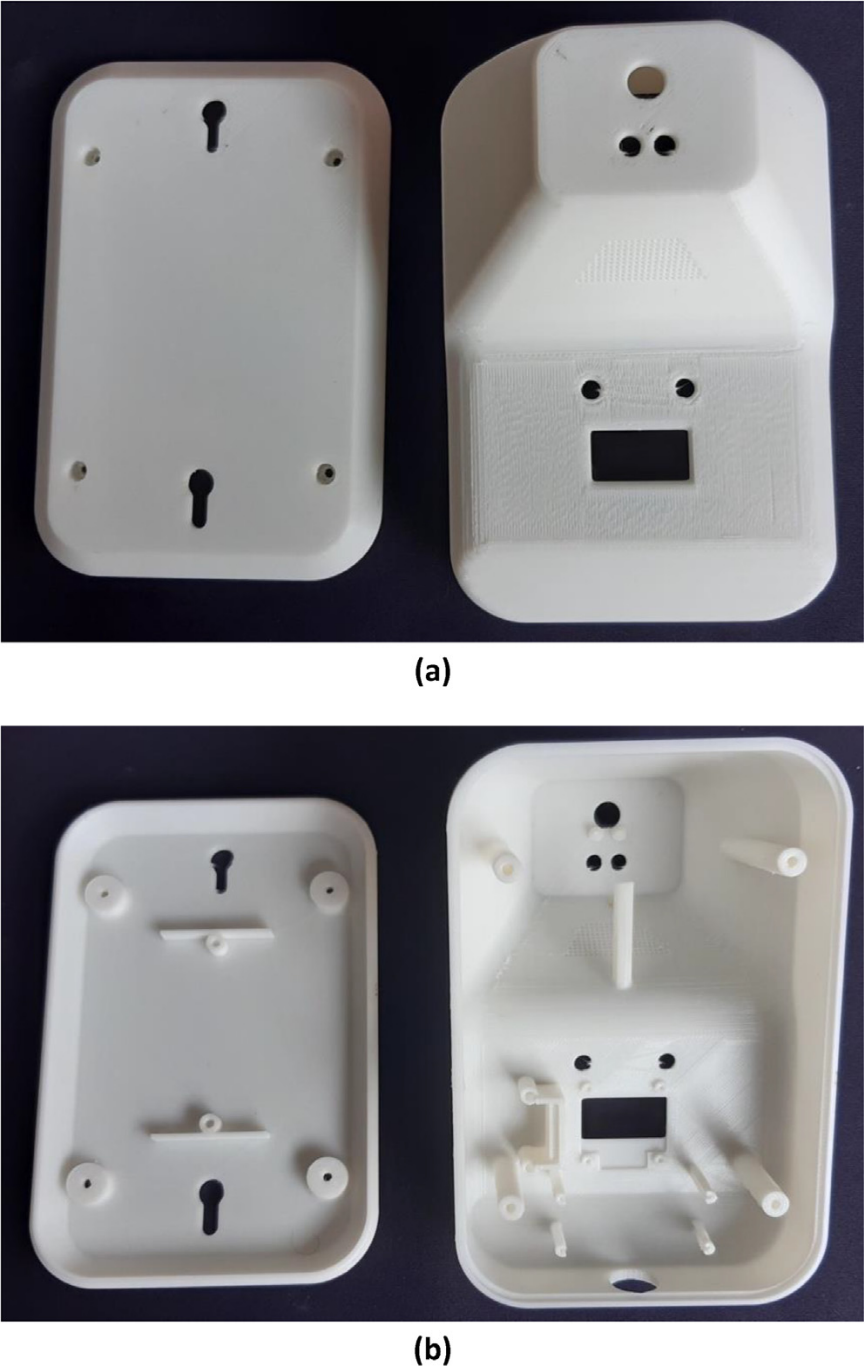
Front case and back case of iThermowall. (a) Top view. (b) Bottom view.

Print Front Case (Case1) and Back Case (Case2) using a 3-D printer with infill 30% and layer 0.2 mm. We recommend using PLA + 3D printing filament with a diameter of 1.75 mm. Fig. 2 shows the result of the 3-D print of the iThermowall Front Case and Back Case from the top view and bottom view.

### 5.2 Uploading the firmware

Before connecting the cable to Arduino Nano, Arduino Firmware need to upload it to the Arduino Nano MCU. To upload the Arduino firmware, please use the following procedure:

1. Open firmware program Arduino_Firmware.ino using Arduino IDE.
2. Connect Arduino Nano to the computer that already installed Arduino IDE, using a mini-B USB cable.
3. On the Arduino IDE, choose the Arduino Nano board, ATmega328P processor, and correct USB port.
4. Verify the program. If there are errors in verification of the library, please install libraries OLED (Adafruit_SSD1306.h) [26], MLX90614 (Adafruit_MLX90614.h) [27], and millisDelay (millisDelay.h) [28].
5. Upload the firmware program. If the upload is successful, there is a message “Done Uploading” on the Arduino IDE.

### 5.3 Component connection of iThermowall

**Fig. 3:**
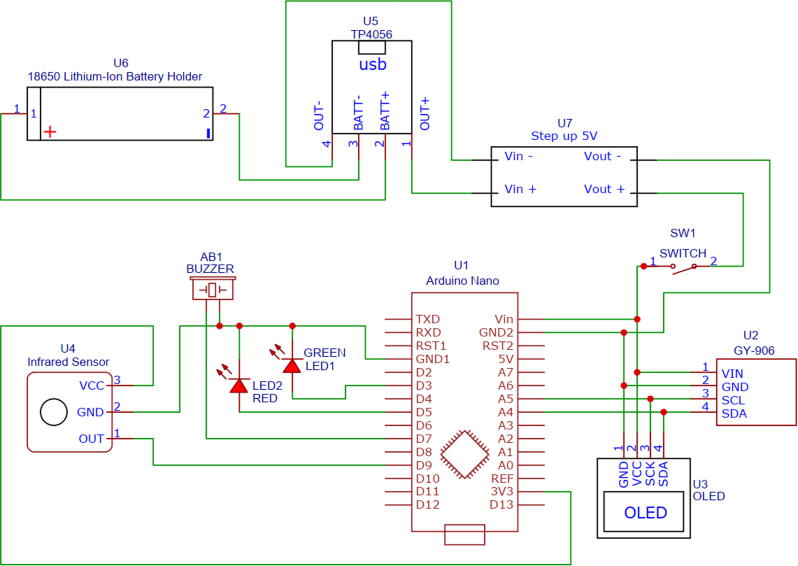
Schematic circuit of the non-contact infrared thermometer wall.

After successfully uploading the firmware, connect the component with the cable, according to Fig. 3 and Table 1.

**Table 1:** Circuit pin connections.

Arduino Nano (U1)	GY-906 (U2)	OLED (U3)	IR sensor (U4)	TP4056 (U5)	Battery Holder (U6)	Step-up 5 V (U7)	Switch (SW1)	GREEN LED (LED1)	RED LED (LED2)	Buzzer (AB1)
*V_in_*	*V_in_*	*VCC*	*–*	*–*	*–*	*–*	*1*	*–*	*–*	*–*
*GND1*	*–*	*–*	*GND*	*GND*	*–*	*–*	*–*	*Cathode*	*Cathode*	*Cathode*
*GND2*	*GND*	*GND*	*–*	*–*	*–*	*V_out_-*	*–*	*–*	*–*	*–*
*A5*	*SCL*	*SCK*	*–*	*–*	*–*	*–*	*–*	*–*	*–*	*–*
*A4*	*SDA*	*SDA*	*–*	*–*	*–*	*–*	*–*	*–*	*–*	*–*
*D3*	*–*	*–*	*–*	*–*	*–*	*–*	*–*	*Anode*	*–*	*–*
*D5*	*–*	*–*	*–*	*–*	*–*	*–*	*–*	*–*	*Anode*	*–*
*D7*	*–*	*–*	*–*	*–*	*–*	*–*	*–*	*–*	*–*	*Anode*
*D9*	*–*	*–*	*OUT*	*–*	*–*	*–*	*–*	*–*	*–*	*–*
*3 V3*	*–*	*–*	*VCC*	*–*	*–*	*–*	*–*	*–*	*–*	*–*
*–*	*–*	*–*	*–*	*BATT+*	*Possitive*	*–*	*–*	*–*	*–*	*–*
*–*	*–*	*–*	*–*	*BATT-*	*Negative*	*–*	*–*	*–*	*–*	*–*
*–*	*–*	*–*	*–*	*OUT+*	*–*	*V_in_+*	*–*	*–*	*–*	*–*
*–*	*–*	*–*	*–*	*OUT-*	*–*	*V_in_−*	*–*	*–*	*–*	*–*
*–*	*–*	*–*	*–*	*–*	*–*	*V_out_+*	*2*	*–*	*–*	*–*

**Fig. 4:**
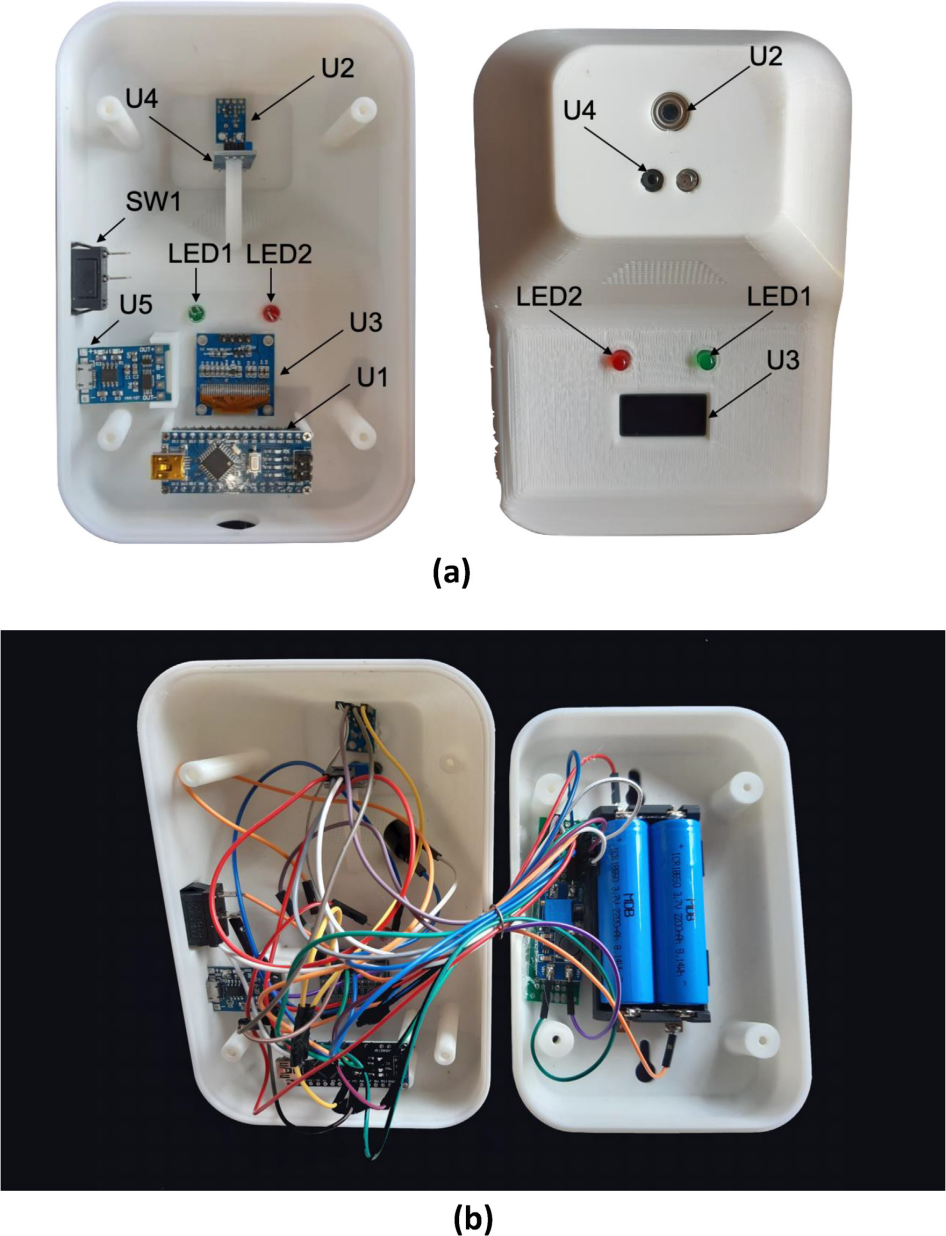
(a) Before connection and (b) after connection of the component to the casing of iThermowall.

Fig. 4 shows the component connections on the inside of iThermowall before and after component connection. Designator's name is shown in Fig. 3.

**Fig. 5:**
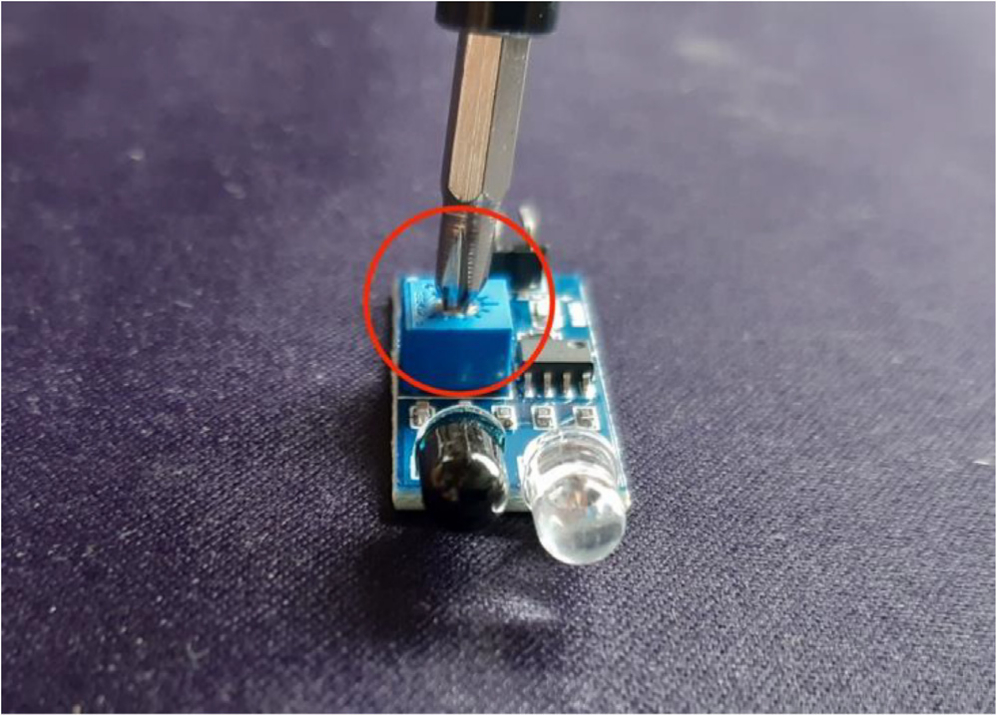
How to set the detection distance of the proximity sensor (U4).

Fig. 5 shows how to set the detection distance from proximity sensor to object. We can use screwdriver to rotate potentiometer on the proximity sensor (U4), so that the detection distance is approximately 50 mm. Rotating potentiometer to clockwise will increase detection distance and counter-clockwise will reduce the detection distance. When setting the proximity sensor (U4), please connect VCC (U4) to the 3 V3 (U1) and GND (U4) to GND (U1).

## 6 Operation instructions

The following is the operation instruction on how to use the iThermowall.

1. Attach two lithium-ion 18,650 batteries to the battery holder in the parallel. Please pay attention to the positive and negative of the battery polarity. Positive battery polarity (U6) connects to B + TP4056 (U5) and negative battery polarity (U6) connects to B- TP4056 (U5).
2. Close the front and back case of the iThermowall using 4 screws of M3 × 10 mm.
3. Turn-on the switch on the right side.
4. The OLED screen will turn-on and show “Initializing.” When the OLED shows “OLED Display allocation failed,” there is something incorrect with the OLED.
5. Put the hardware on the wall approximately 150 cm (close to the forehead), as shown in Fig. 6.

**Fig. 6:**
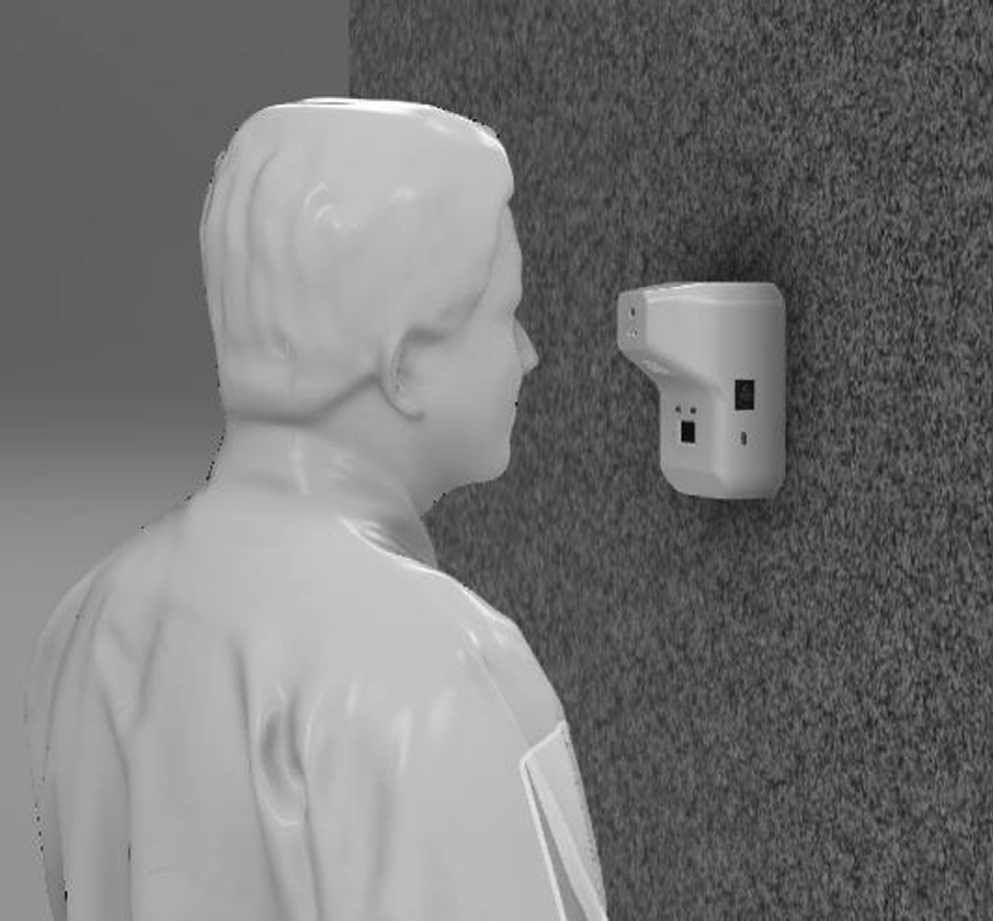
Position of the iThermowall.
6. If the battery starts to run out of energy, charge the hardware using a power adapter charger 5 V DC with a micro-USB port.

## 7 Validation and characterization

### 7.1 Temperature measurement validation

For validation, the iThermowall sensed the body temperature in various sites, they were head, brow, ear, and wrist, and compared by a reference thermometer. The reference thermometer we used was a handheld infrared thermometer, SK-T008 (Luzhou Skinod Technology, China) with accuracy ± 0.2 °C within range 35.0 °C − 42.0 °C at operating temperature in 10–40 °C [29]. Volunteers were recruited in this study, including men and women with age ranges from 3 to 40 years old. Oral informed consent was obtained from the volunteers or parents of the children after explaining the purpose of temperature measurement. Simultaneous temperature measurements were conducted in the morning, afternoon, and night, with the head, forehead, external auditory canal (EAC), and wrist as the target points. The measurements were performed by comparing the iThermowall to the reference thermometer. Briefly, the probe of the infrared thermometer was directed into the head, forehead, EAC, and wrist. The investigation was held in this position for a few seconds until the device bleeped and display the temperature in Celsius degree. The measurements performed from each point were repeated three times. Table 2, Table 3 show the comparison between the reference thermometer and iThermowall.

**Table 2:** ANOVA analysis.

*Source of Variation*	*SS*	*df*	*MS*	*F*	*P-value*	*F crit*
Between Groups	2.634861	3	0.878287	2.579664	0.060656	2.739502
Within Groups	23.15167	68	0.340466			
Total	25.78653	71

**Table 3:** *T*-test analysis.

Sites		Reference	iThermowall
Head	Mean	36.19444	36.05555556
	Variance	0.013497	0.368496732
	Observations	18	18
	Hypothesized Mean Difference		0
	df		18
	t Stat		0.953401
	P(T ≤ t) one-tail		0.176508
	t Critical one-tail		1.734064
	P(T ≤ t) two-tail		0.353016
	t Critical two-tail		2.100922

Brow	Mean	36.41111	36.56111111
	Variance	0.026928	0.16369281
	Observations	18	18
	Hypothesized Mean Difference		0
	df		22
	t Stat		−1.45761
	P(T ≤ t) one-tail		0.079537
	t Critical one-tail		1.717144
	P(T ≤ t) two-tail		0.159075
	t Critical two-tail		2.073873

EAC	Mean	36.42222	36.34444444
	Variance	0.114771	0.413202614
	Observations	18	18
	Hypothesized Mean Difference		0
	df		26
	t Stat		0.454136
	P(T ≤ t) one-tail		0.32675
	t Critical one-tail		1.705618
	P(T ≤ t) two-tail		0.6535
	t Critical two-tail		2.055529

Wrist	Mean	36.3	36.16666667
	Variance	0.015294	0.416470588
	Observations	18	18
	Hypothesized Mean Difference		0
	Df		18
	t Stat		0.860897
	P(T ≤ t) one-tail		0.200309
	t Critical one-tail		1.734064
	P(T ≤ t) two-tail		0.400617
	t Critical two-tail		2.100922

Testing the ANOVA, we found the P-value is greater than the considerable level of significance (α = 0.05), indicating no differences for all means among sites detected by the iThermowall. Then we conducted T-test analysis, the P-values for all the test is greater than 0.05, means that the mean Celsius temperature for both groups (Reference and iThermowall) for all sites of the sensor are similar. The difference between the mean Celsius temperature for both groups in fours site were less than 0.2 °C. There are several factors influencing the accuracy of the iThermowall, such as ambient temperature, the alignment of the thermometer, non-linearity, target emissivity value, and background radiation [30], [31]. The iThermowall used the GY-906 sensor module that utilized an MLX90614 temperature sensor with accuracy ± 0.2 °C for a limited temperature range around the human body temperature [21].

### 7.2 Green and Red LED validation

**Fig. 7:**
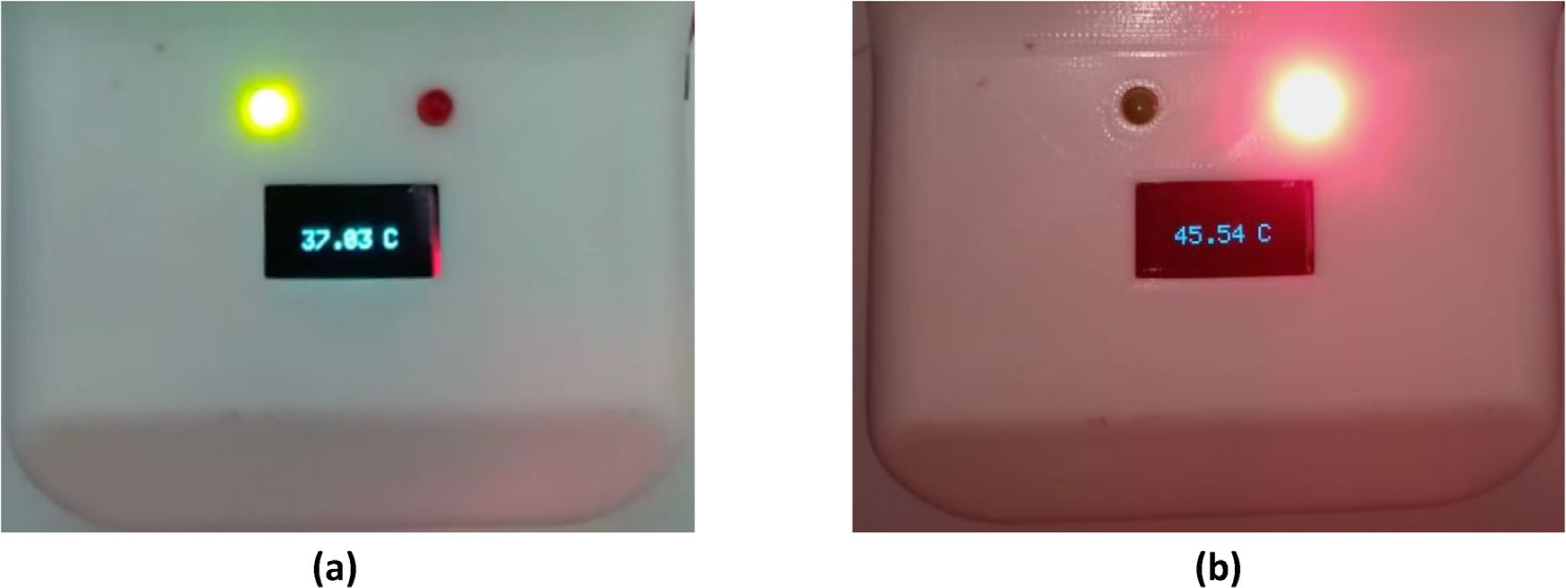
(a) Green LED turn on when the temperature is less than 38 °C. (b) Red LED turn on when the temperature is more than 38 °C. (For interpretation of the references to colour in this figure legend, the reader is referred to the web version of this article.)

In the iThermowall algorithm, Green LED and active buzzer turn on for 1 s when the temperature is less than 38 °C. On the other hand, Red LED and active buzzer turn on for 5 s when the temperature is more than 38 °C. Fig. 7(a) and 7(b) show the validation of the Green and Red LED to the temperature, respectively. In Fig. 7(a), the Green LED is turn on when the measured temperature is 37.03 °C (less than 38 °C). Fig. 7(b) shows that the Red LED is turned on when the measured temperature is 45.54 °C (more than 38 °C).

### 7.3 Power consumption and battery life

**Fig. 8:**
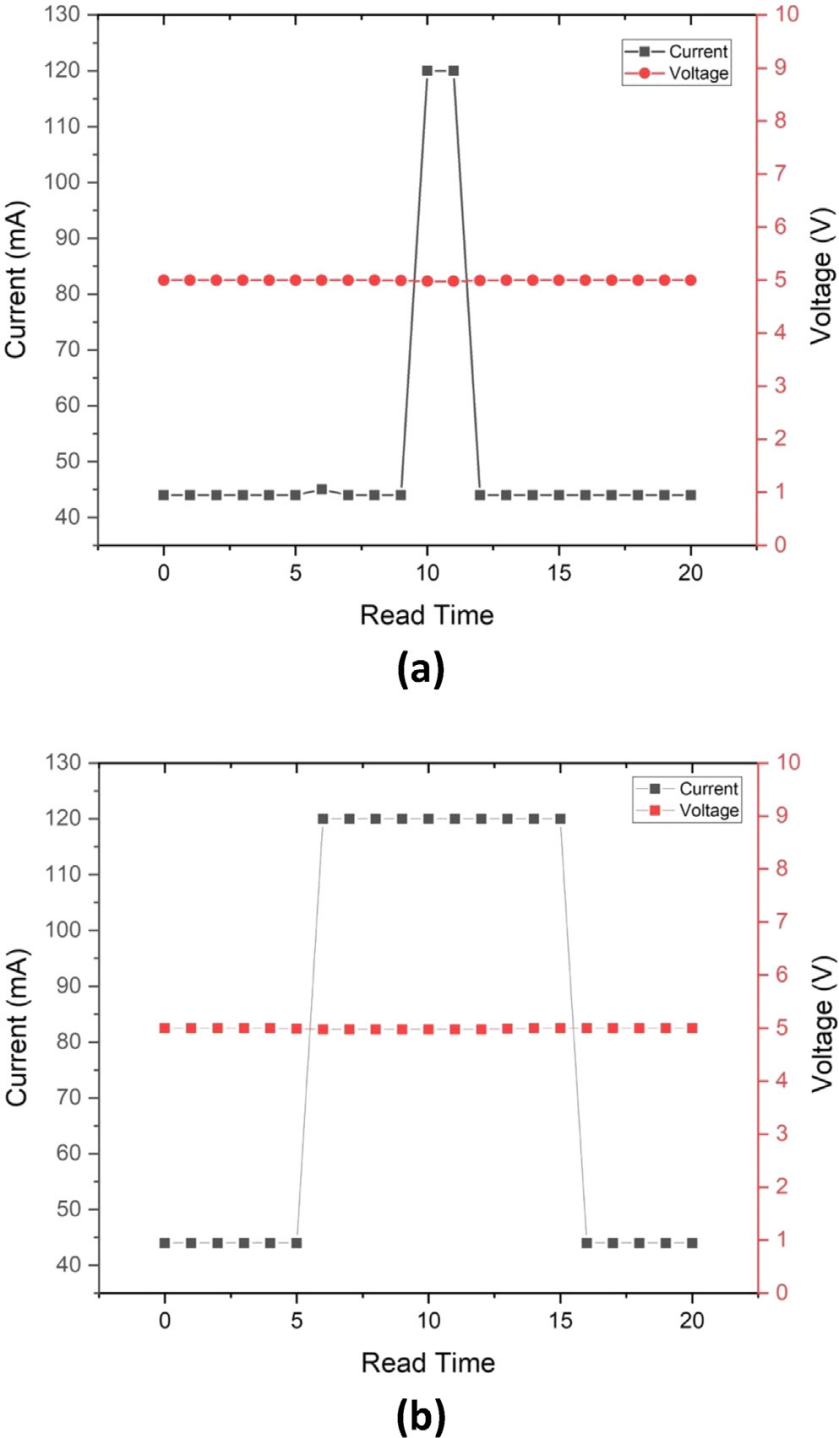
I-V (Current-Voltage) characterization of the iThermowall when the temperature is (a) less than 38 °C and (b) more than 38 °C.

The power characterization of iThermowall was conducted by USB Meter UM24 (RuiDeng Tech., China) with voltage and current measurement resolution of 0.01 V and 0.001 A, respectively. Fig. 8(a) and (b) show the I-V (Current-Voltage) characterization of the thermometer when the temperature is less than 38 °C and more than 38 °C, respectively. The period of the current peak in Fig. 8(b) is longer than Fig. 8(a) due to the longer period of Red LED and Buzzer turn on (5 s) when the temperature is more than 38 °C. In a temperature less than 38 °C, the Green LED and Buzzer only turn on for 1 s.

Fig. 8 shows that the maximum current consumption during LED and Buzzer turn on (when the sensor detects people in front of the iThermowall) is 118 mA. On the other hand, minimum current consumption during LED and Buzzer turn off (when the sensor does not detect people in front of the iThermowall) is 44 mA. The voltage is stable at around 4.98 V to 5.00 V.

To measure the power consumption of the iThermowall, the total current is assumed by 30% for the maximum current and 70% for the minimum current.

$$W=\left(V\times 30\%I_{\max}\right)+\left(V\times 70\%I_{\min}\right)=\left(5\text{V}\times 30\%\times 118\text{mA}\right)+\left(5\text{V}\times 70\%\times 44\text{mA}\right)=331\text{mW}$$

where *W* is power consumption, *V* is voltage, *I_max_* is maximum current, and *I*_min_ is minimum current. The energy of the two lithium-ion batteries was calculated by 80% of the total energy.

$$E_{\mathrm{bat}}=80\%\times 2\times 3.7V\times 2200\text{mAh}=13024\text{mWh}$$

where *E_bat_* is battery energy. From the battery energy and power consumption, we can calculate battery lifetime.

$$T_{\mathrm{bat}}=\left(E_{\mathrm{bat}}\right)/\left(W\right)=\left(13024\text{mWh}\right)/\left(331\text{mW}\right)=39.3\text{h}$$

where *T_bat_* is battery lifetime. From the calculation, the iThermowall will work without charging up to 39.3 h. On the other hand, the iThermowall can continue operating while the hardware is charging because of Lithium Battery Charger Module TP4056. Therefore, to support its continuous operation, we recommend having one additional power bank for each iThermowall unit.

### 7.4 Performance comparison

There are a number of thermometer types that have their own criteria in detecting the human body temperature. Measuring techniques, target distance, accuracy, power supply, and price become essential concerns to develop a new device since the device's compatibility should be suitable to needed. Here, we provide the specifications of iThermowall compared to other thermometers, as shown in Table 4.

**Table 4:** Performance comparison.

Thermometer type	Temperature measurement	Operational distance	Accuracy	Dimension and weight	Power supply	Cost of hardware
iThermowall (This work)	Automatic	50 mm	±0.2 °C	157 mm × 102 mm × 97.5 mm and 0.6 kg	Rechargeable Lithium-ion Battery 18,650	$35 USD
Handheld Infrared Thermometer [29]	Manual	50–150 mm	±0.2 °C	100 mm × 46 mm × 160 mm and 0.4 kg	Non-rechargeable DC 9 V Battery	$25 USD
Infant Body Temperature [15]	Manual	0 mm (need contact)	±0.5 °C	–	Rechargeable Lithium-ion Polymer Battery	–
Real-time patient Temperature [16]	Manual	0 mm (need contact)	±0.5 °C	–	USB Power Adapter Charger 5 V DC, 5 W	$61 USD
FLIR Thermovision [23], [25]	Automatic	>2000 mm	±0.2 °C	216 mm × 73 mm × 75 mm and 0.9 kg	12–24 V DC, 40 W	$16,000 USD
OptoTherm Thermoscreen [24], [25]	Automatic	>2000 mm	±0.2 °C	355 mm × 180 mm × 125 mm and 5 kg	100/240 VAC 50/60 Hz	$22,000 USD

## CRediT authorship contribution statement

**Tomy Abuzairi:** Conceptualization, Supervision, Writing - original draft, Writing - review & editing, Visualization. **Nur Imaniati Sumantri:** Formal analysis, Validation, Investigation, Data curation, Writing - original draft, Writing - review & editing. **Ahli Irfan:** Concentualization, Formal Analysis, Methodology, Visualization. **Ridho Maulana Mohamad:** Software, Methodology.

## Declaration of Competing Interest

The authors declare that they have no known competing financial interests or personal relationships that could have appeared to influence the work reported in this paper.
